# Supplementary material for: Notch3-dependent β-catenin signaling mediates EGFR TKI drug persistence in EGFR mutant NSCLC
Source: Nat Commun. 2018 Aug 10;9:3198. doi: 10.1038/s41467-018-05626-2 (PMC6090531; doi:10.1038/s41467-018-05626-2)
Supplement: Supplementary file 1 — Supplementary Information [file 41467_2018_5626_MOESM1_ESM.pdf]

Notch3-dependent β-catenin signaling mediates EGFR TKI drug persistence in EGFR mutant NSCLC

Arasada et al.

Supplementary Information

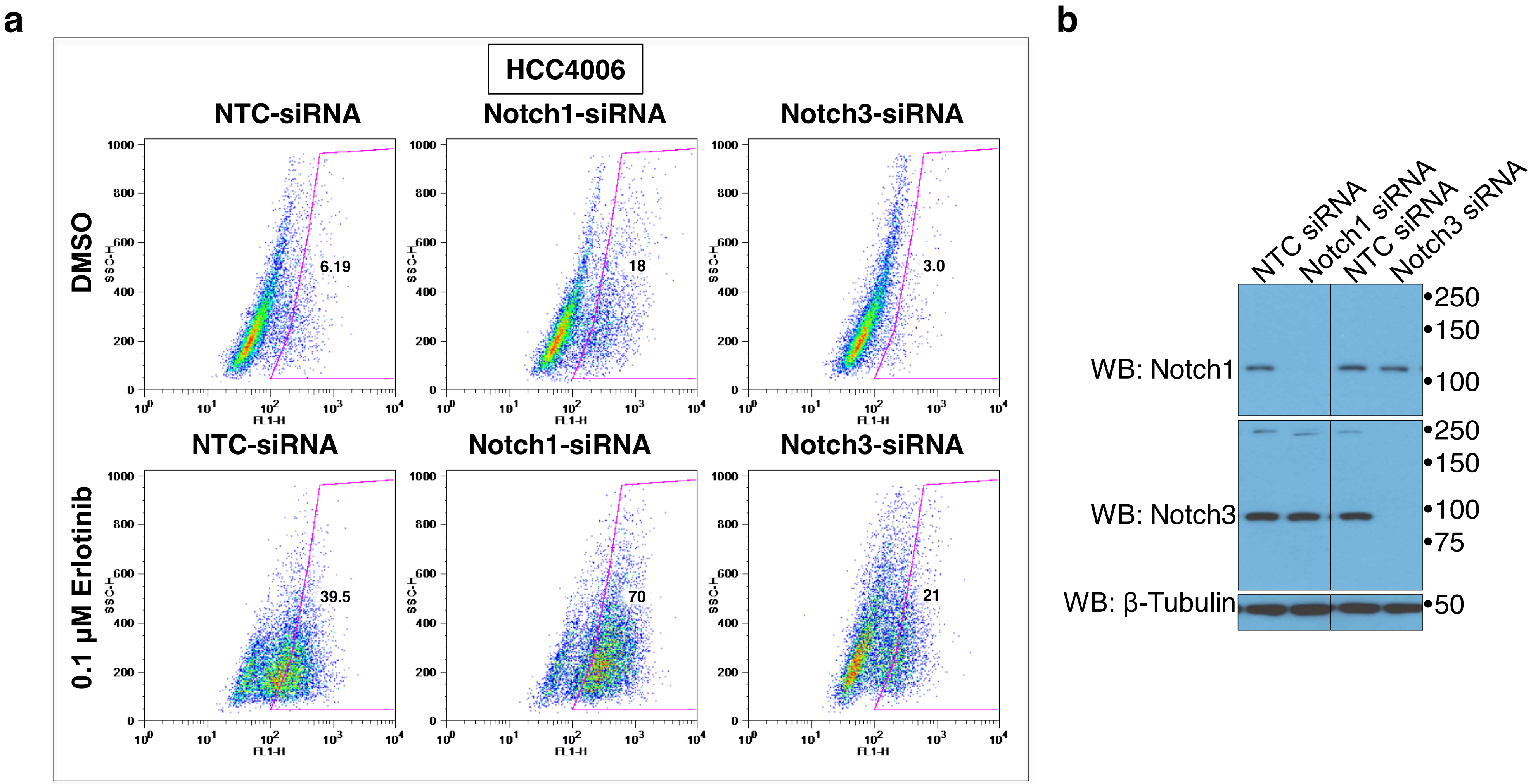

Supplementary Figure 1 Differential role of Notch1 and Notch3 in EGFR TKI induced persistent cells in EGFR mutant NSCLC. **a** Analysis of ALDH activity in HCC4006 cells treated with DMSO or erlotinib in combination with various siRNAs; non-targeting control (NTC), Notch1 and Notch3. **b** Western analysis showing the specificity of Notch1 and Notch3 knockdown.

HCC4006

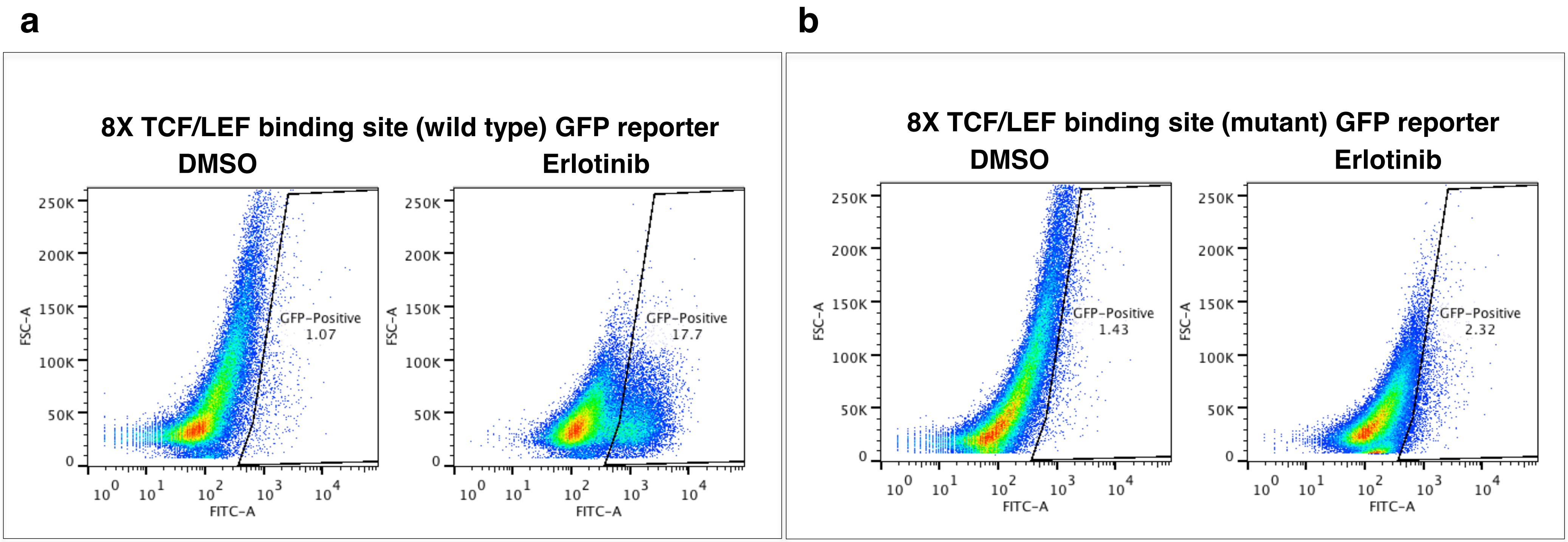

Supplementary Figure 2 EGFR TKI treatment induces transcriptional activity of β-catenin. HCC4006 cells expressing TCF-GFP wild type reporter- Tcf/Lef **a** or TCF-GFP mutant reporter **b** were treated with DMSO or 0.1 μM erlotinib for 6 days and FAC Sorted for GFP fluorescence. Representative FACS analysis of reporter activity is presented in histograms where GFP-positive fraction is represented in the box.

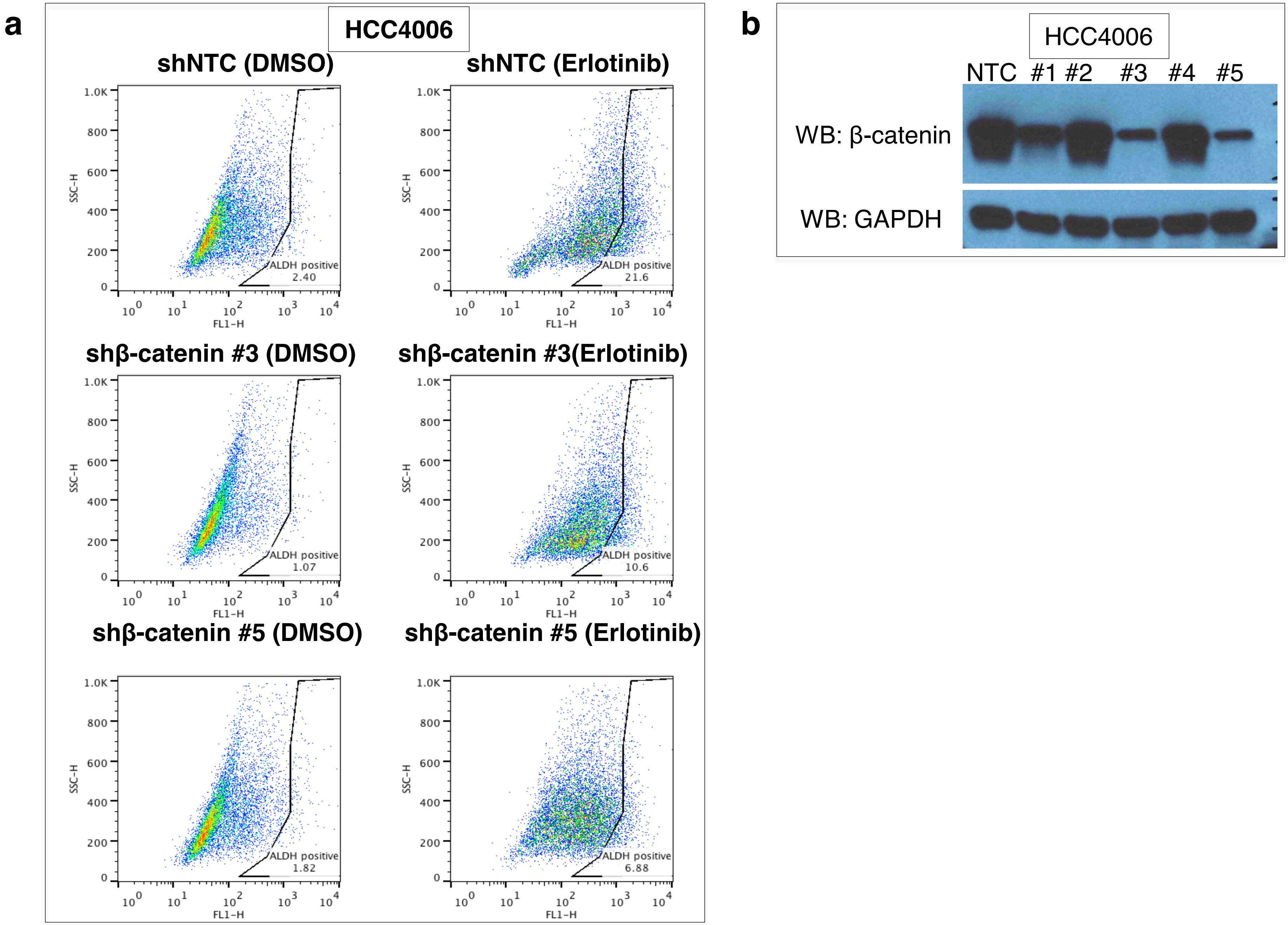

Supplementary Figure 3 Effect of β-catenin knockdown on EGFR TKI induced ALDH positive cells. HCC4006 cells were stably infected with shRNAs that included non-targeting control (shNTC) or five individual shRNAs for β-catenin (shRNAs #1-#5). Cells were treated with DMSO or erlotinib and subjected to ALDH assay. **a** Representative ALDH FACS analysis and **b** western analysis β-catenin knockdown are shown.

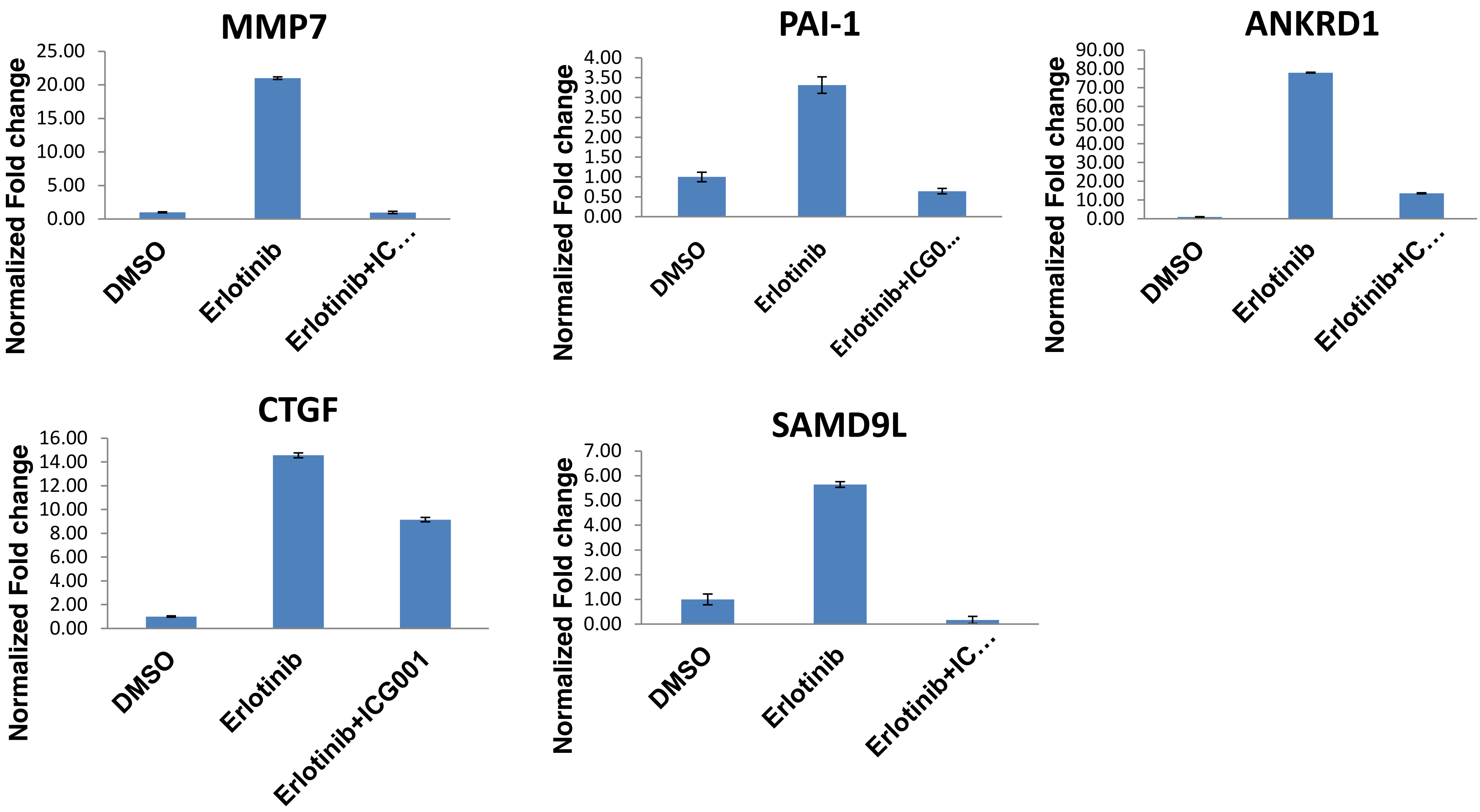

Supplementary Figure 4 EGFR TKI therapy induced β-catenin signaling targets are sensitive to β-catenin inhibitor, ICG-001. HCC827 xenograft tumors were treated with control, erlotinib or combination of erlotinib and ICG-001. Total RNA was isolated from remaining tumor after the treatment and qRT-PCR analysis was performed on β-catenin transcriptional targets. Error bars represent SD from technical triplicates.

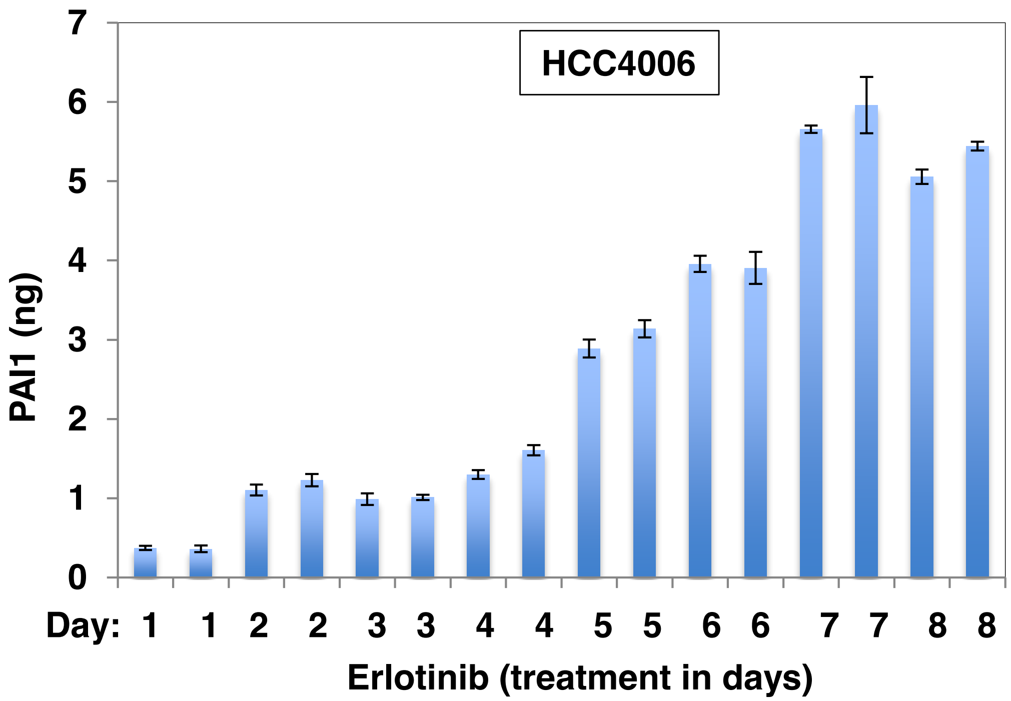

Supplementary Figure 5 EGFR TKI treatment induces secretion of PAI1 in HCC4006 cells. PAI1 ELISA demonstrating that erlotinib treatment induces PAI1 levels in HCC4006. Cells were treated with erlotinib or DMSO for various time periods and soluble PAI1 was measured from cell culture supernatant using PAI1 ELISA. Error bars represent SD from technical triplicates from two different experiments.

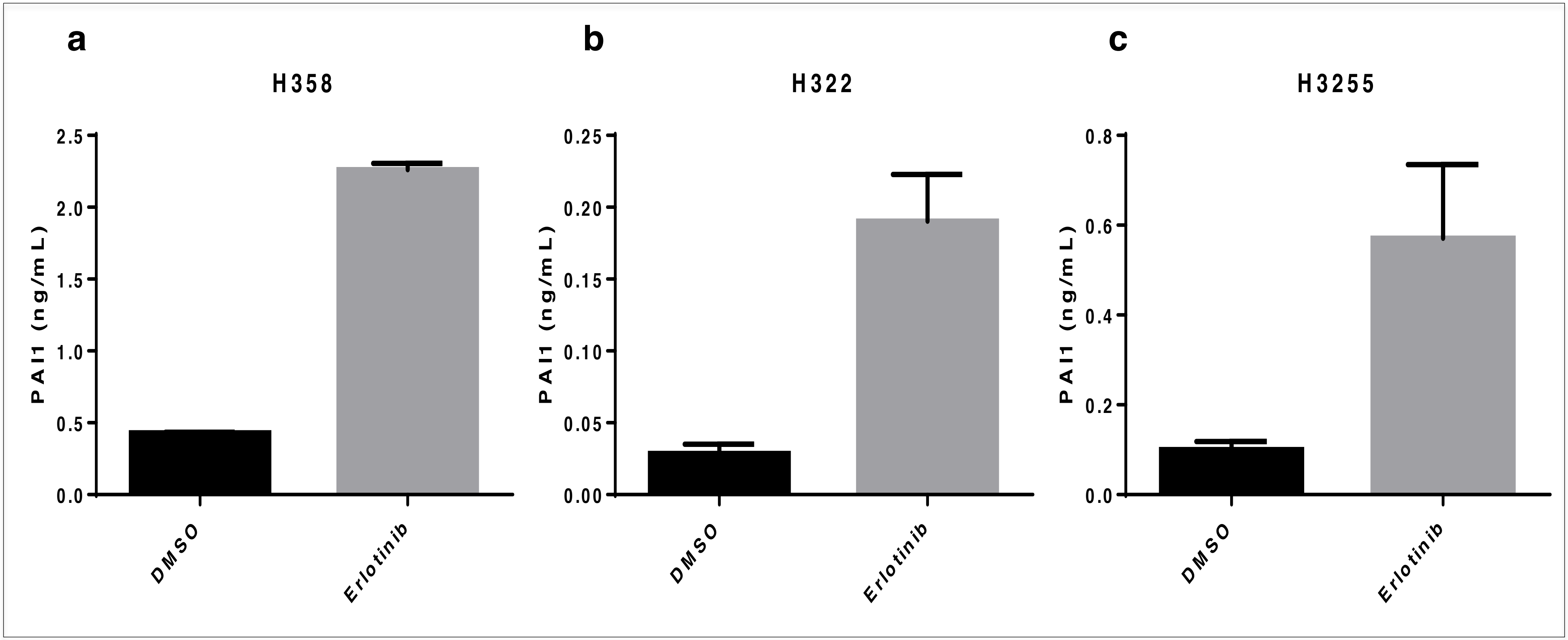

Supplementary Figure 6 EGFR TKI treatment induces secretion of PAI1 in various EGFR mutant and wild type NSCLC cell lines that are sensitive to EGFR TKI. PAI1 ELISA demonstrating that erlotinib treatment induces PAI1 levels. **a** H358, **b** H322 and **c** H3255 cells were treated with erlotinib or DMSO for 6 days and soluble PAI1 was measured from cell culture supernatant using PAI1 ELISA. Error bars represent s.e.m from technical triplicates.

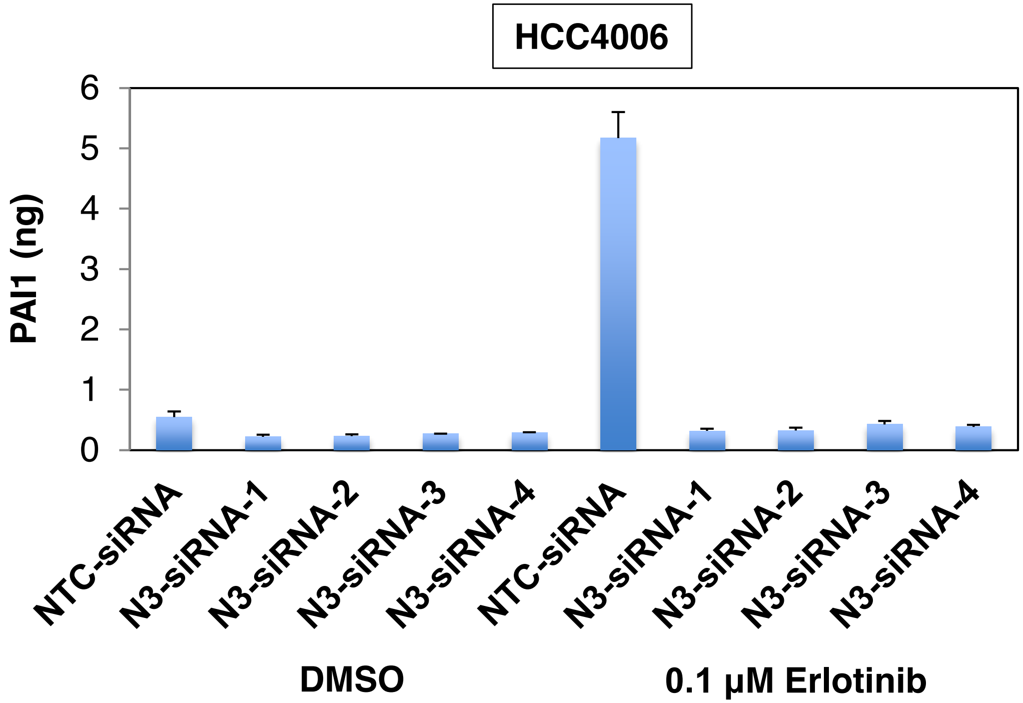

Supplementary Figure 7 EGFR TKI treatment induced secretion of PAI1 requires Notch3. ELISA analysis of PAI1 levels in HCC4006 cells. HCC4006 cells were treated with non-targeting control siRNA (NTC) or four different siRNAs targeting Notch3 in combination with erlotinib or vehicle control (DMSO) treatment. Error bars represent SD from technical triplicates.

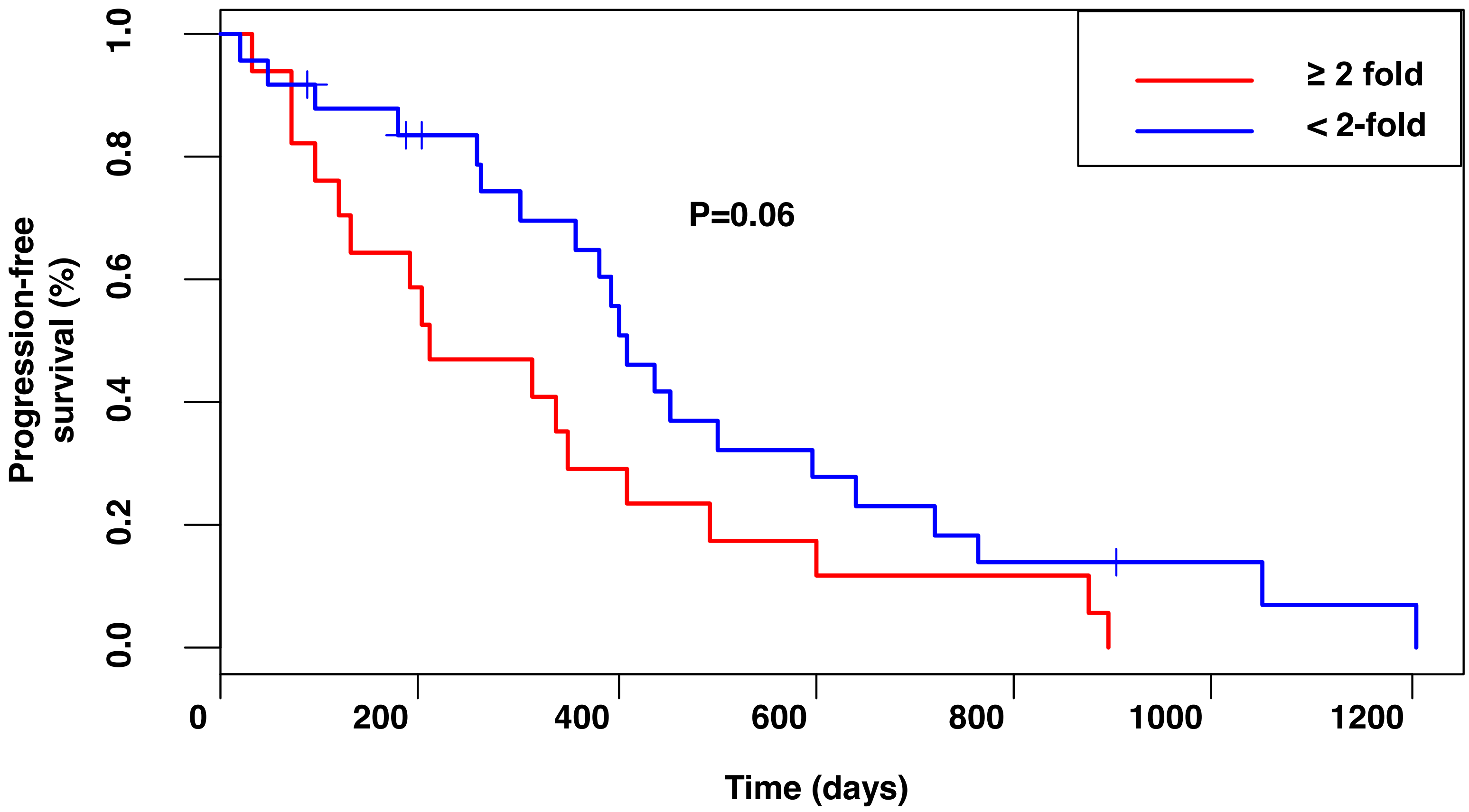

Supplementary Figure 8 EGFR TKI therapy induces secretion of PAI1 in patient serum and predicts EGFR TKI resistance in EGFR mutant lung cancer. Patients were dichotomized by 2- fold change in PAI1 levels after treatment as high (2-fold change or higher) and low (less than 2-fold change) risk groups. Progression-free survival (PFS) of patients treated with EGFR TKI therapy over the period was estimated by the Kaplan-Meier method. The log-rank test was used to compare the progression-free survival (PFS) of the two groups. Patients with high PAI1 levels after the EGFR TKI therapy group led to a worse PFS compared to the low PAI1 levels group (P=0.068, median PFS: 209 days vs. 408 days; HR: 0.55 [95% C.I.: 0.28-1.06])

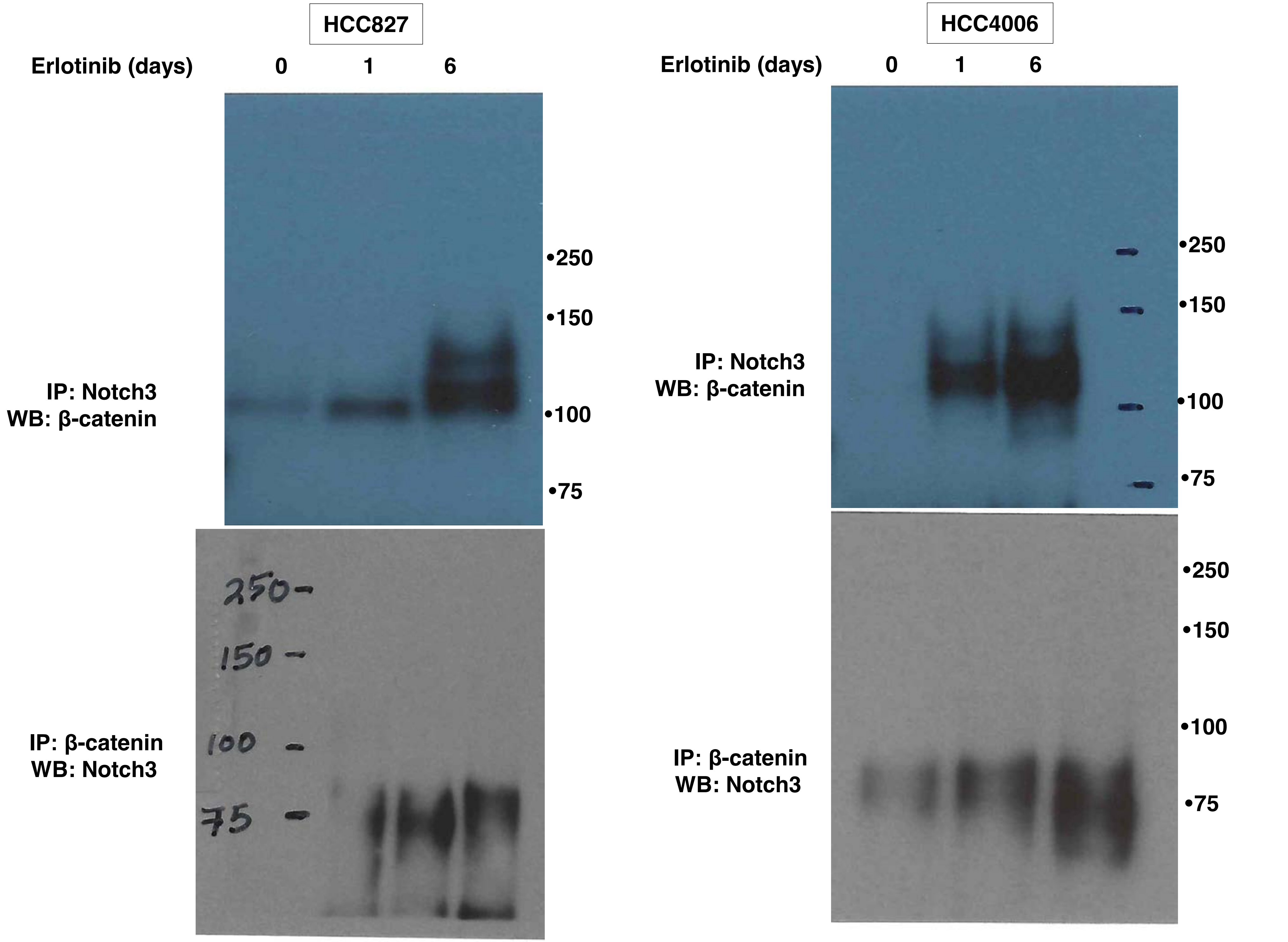

Supplementary Figure 9 Uncropped images of key western blots shown in panels a and b of Figure 3 with molecular weight maker indicated.

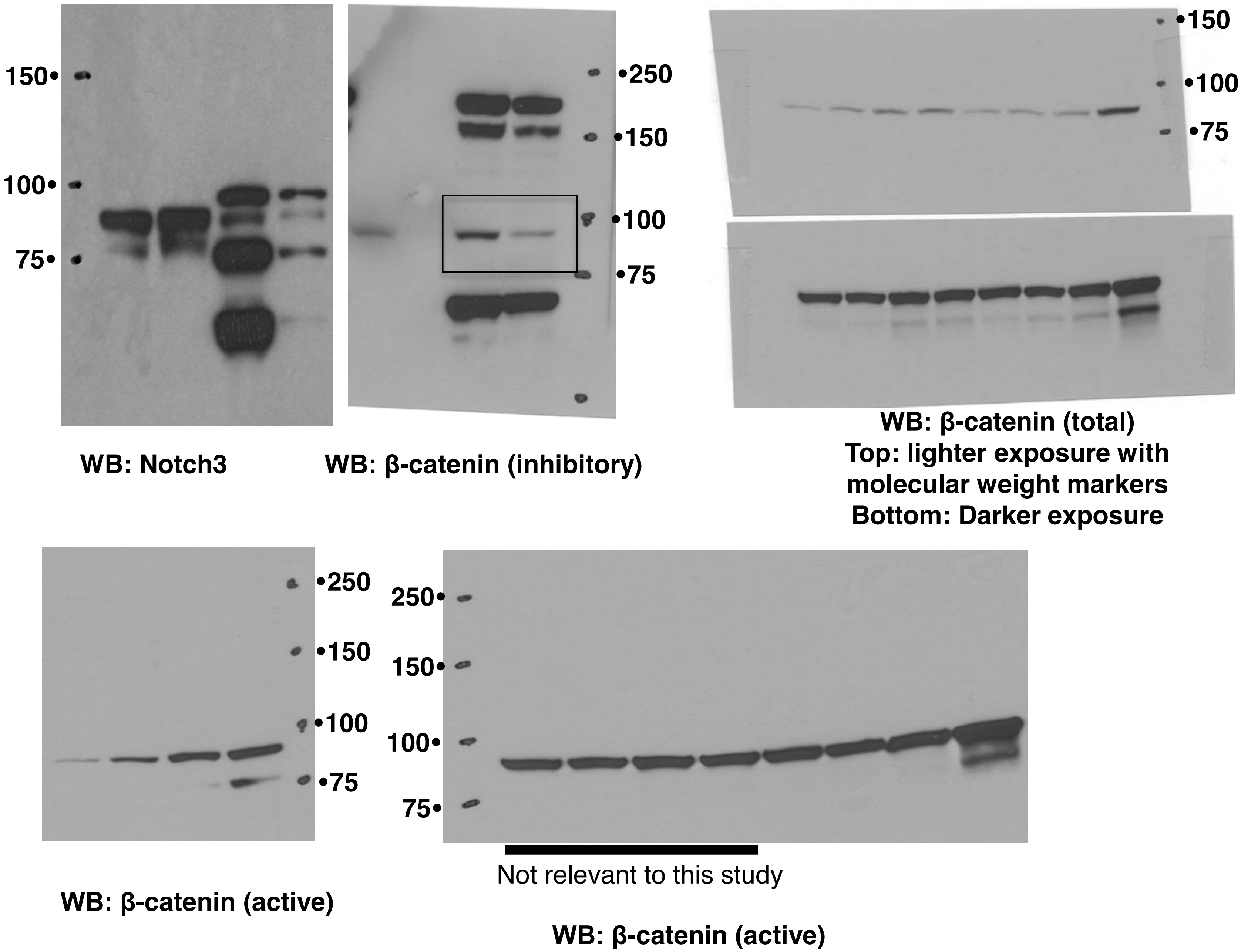

Supplementary Figure 10 Uncropped images of key western blots shown in panels c of Figure 3 with molecular weight maker indicated.

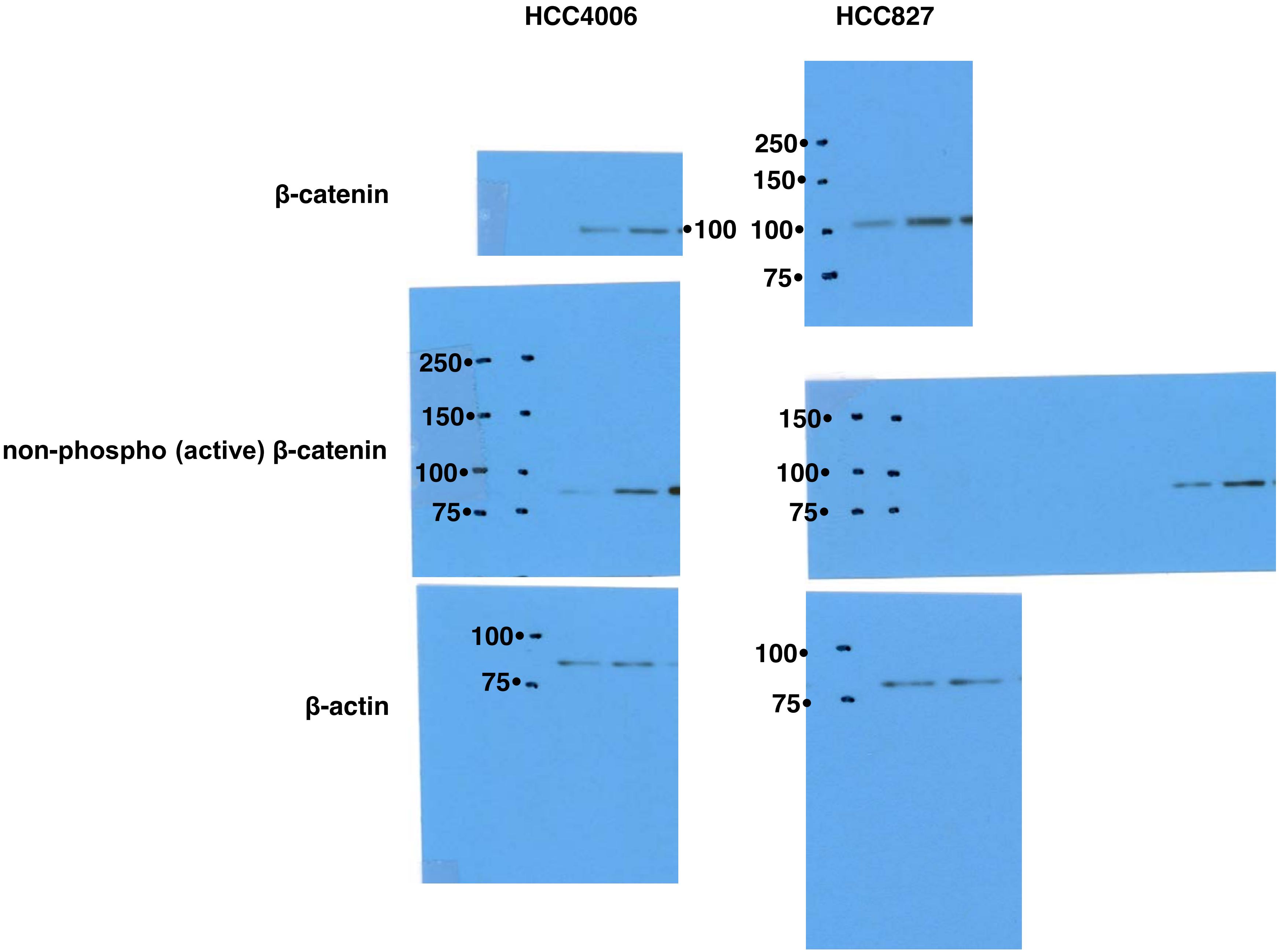

Supplementary Figure 11 Uncropped images of western blots shown in panels a of Figure 4 with molecular weight maker indicated.

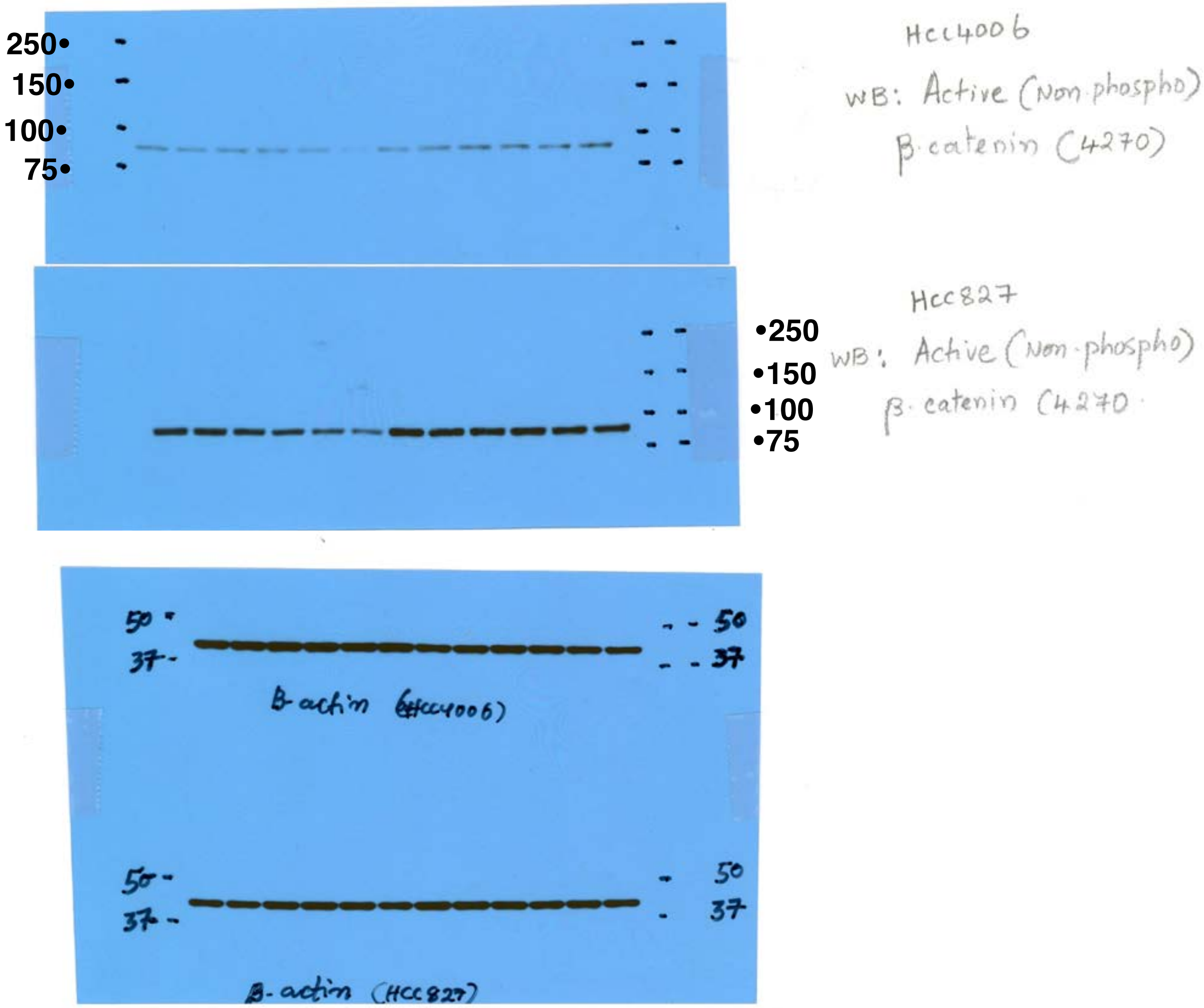

Supplementary Figure 12 Uncropped images of western blots shown in panel b of Figure 4 with molecular weight maker indicated.

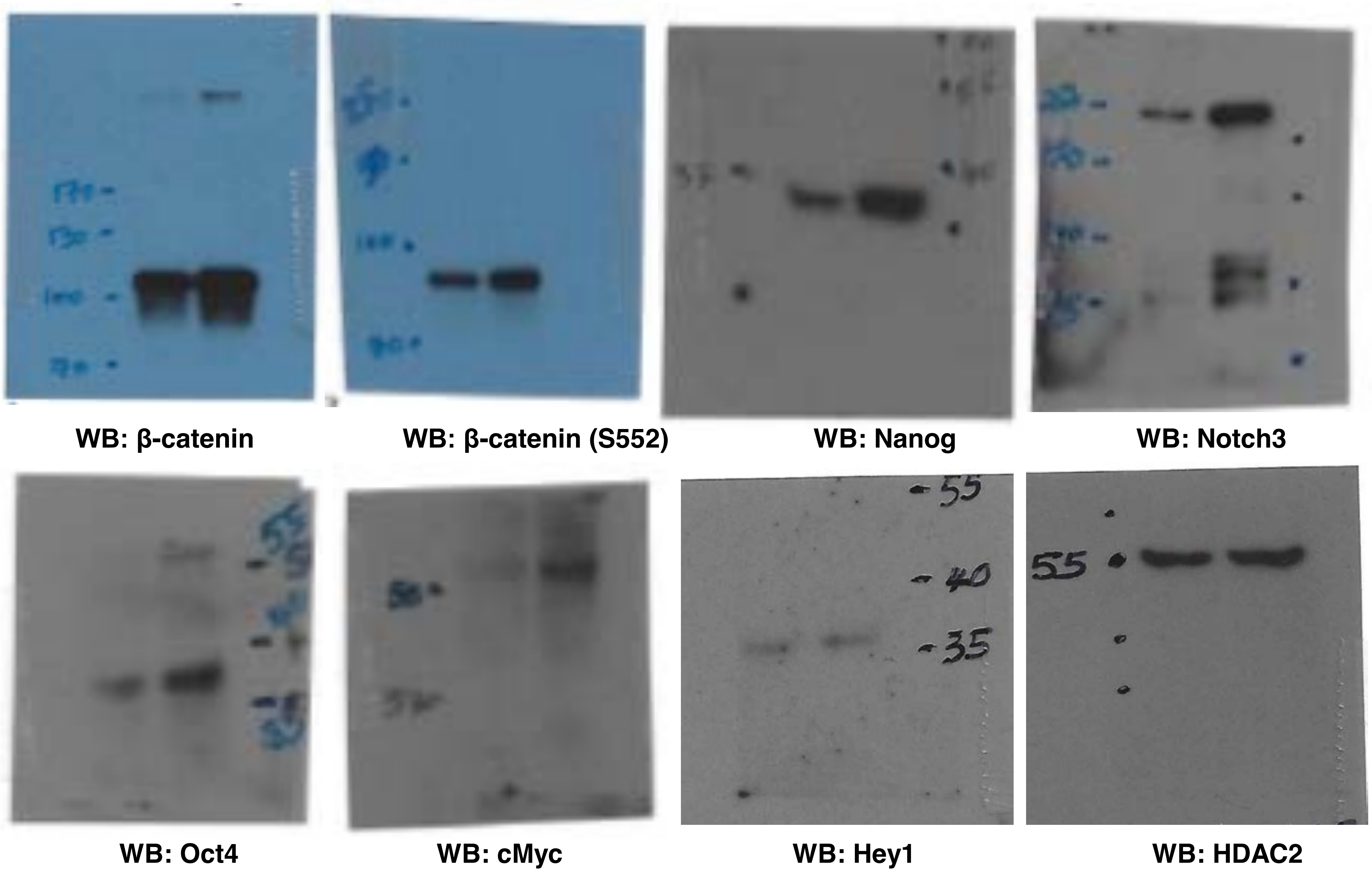

Supplementary Figure 13 Uncropped images of western blots shown in panel d of Figure 4 with molecular weight maker indicated.

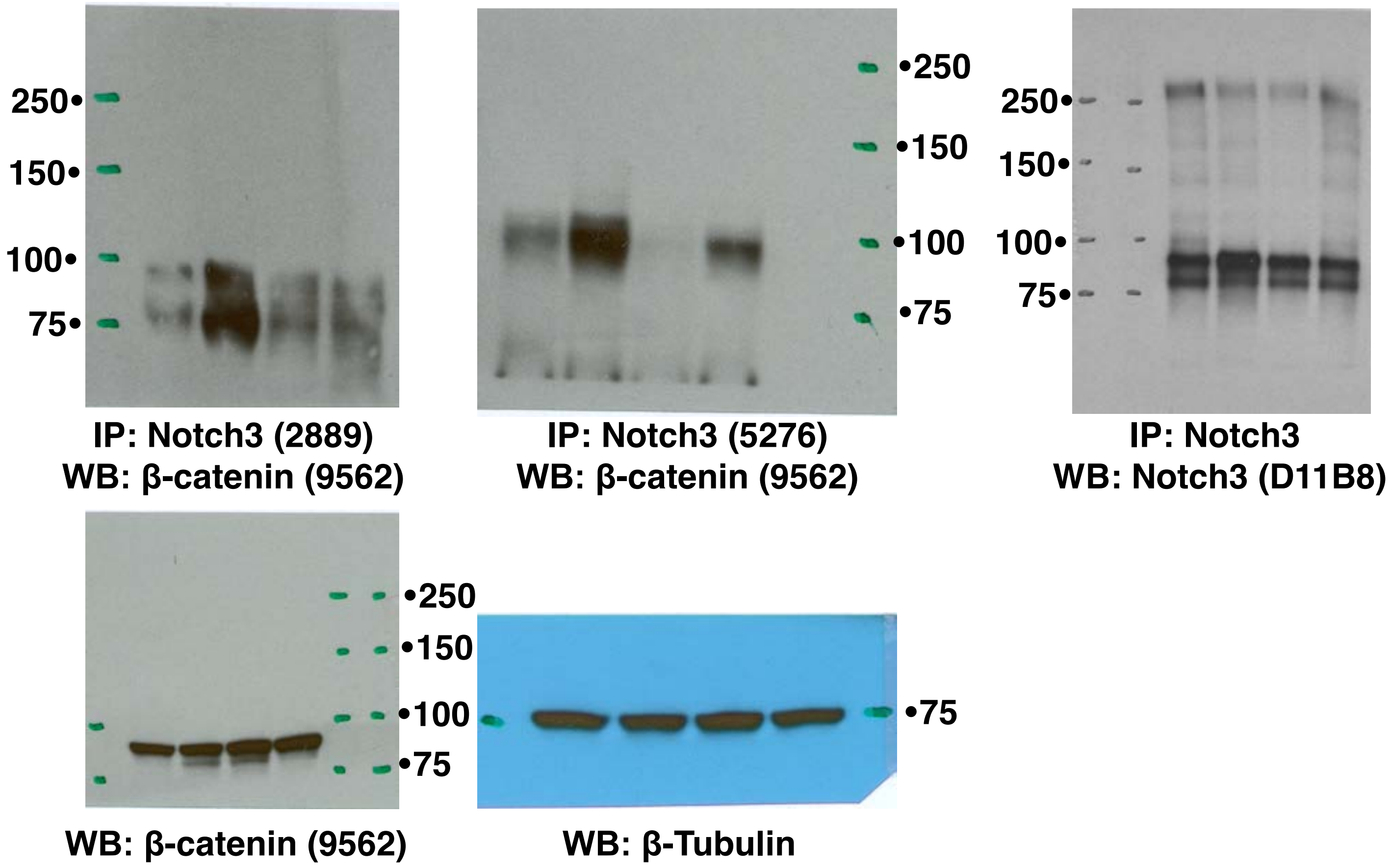

Supplementary Figure 14 Uncropped images of western blots shown in panel a of Figure 5 with molecular weight maker indicated.

Differentially regulated genes in EGFR TKI induced DPCs

	Erlotinib vs. DMSO		Erlotinib+N1 vs Erlotinib		Erlotinib+N3 vs Erlotinib		Ratio differential comparison 1 vs 3
Gene Symbol	Comparison-1 Fold change	P Value	Comparison-2 Fold change	P Value	Comparison-3 Fold change	P Value	
MMP7	6.47	0	1.51	0	-2.03	0	8.5
PAI1	4.72	0	1.37	0	-3.33	0	8.05
RASGRP	4.1	0	1.21	0.0019	-2.96	0	7.06
MIR503	3.54	0	1.22	0.0083	-1.3	0.0011	4.84
EGOT	3.25	0	1.41	0.0086	-1.56	0.0013	4.81
HIST1H	2.97	0	1.33	0.0006	-2.01	0	4.98
SAMD9L	2.82	0	2.02	0	-1.31	0.0001	4.13
IFI27	2.41	0	1.62	0	-1.23	0.0002	3.64
ANKRD1	2.39	0	1.27	0	-1.54	0	3.93
KCNK15	2.26	0	1.32	0.0001	-1.54	0	3.8
GADD45	2.06	0	1.4	0	-1.91	0	3.97
HBEGF	2.06	0	1.26	0	-1.43	0	3.49
ABI3BP	2.05	0	2.22	0	-2.46	0	4.51
CTGF	2.02	0	1.5	0	-2.82	0	4.84
SRPX2	-3.71	0	-1.33	0	1.49	0	-5.2
VCAN	-3.3	0	-1.41	0	2.05	0	-5.35
ZPLD1	-2.99	0	-1.44	0	1.5	0	-4.49
RBL1	-2.7	0	-1.21	0.0019	1.2	0.0016	-3.9
SLITRK	-2.51	0	-1.36	0	1.4	0	-3.91
HLTF	-1.87	0	-1.2	0.0003	1.27	0	-3.14

Supplementary Table 1. Identification of differentially regulated genes in EGFR TKI induced DPCs in EGFR mutant NSCLC cells. We applied three tier selection criteria to identify EGFR TKI induced DPCs. Initially, genes that are upregulated by 2 or more folds or downregulated by 1.5 or more folds for erlotinib

versus DMSO, with P value less than 0.0005 (5 false positives out of 10K genes) are considered as top genes (erlotinib regulated genes) and selected for further analyses. In the second tier selection erlotinib regulated genes were further narrowed based on further increase or decrease in gene expression and follow the same trend as erlotinib after Notch1 knockdown (erlotinib+shNotch1 versus erlotinib alone) which are considered as erlotinib and shNotch1 regulated. In the final selection, we identified genes, with at least 1.2 fold change in the opposite direction compared to erlotinib+shNotch1-regulated genes (erlotinib+siNotch3 versus erlotinib+siNotch1) and considered as DPC regulators. List of genes that are differentially expressed after erlotinib treatment in combination with non-targeting control siRNA (DMSO), Notch1 siRNAs (N1), or Notch3 siRNAs (N3) in HCC4006 cells. Green corresponds to positive regulators and orange corresponds to negative regulators of DPCs. Fold change and p values for each group are presented. List of DPC regulators, positive (green) and negative (red) were sorted based on the high to low fold change.

β-catenin signaling in EGFR TKI DPCs

Gene	Function	Location	Physiological role	Transcriptional role
MMP7	Zinc dependent endopeptidase. Breakdown of extracellular matrix (proteoglycans, fibronectin, elastin and casein).	Extracellular	Metastasis, embryonic development, tissue remodeling	β-catenin
PAI1	Serine (cysteine) peptidase inhibitor (principal inhibitor of tPA and uPA)	Extracellular	Cancer stem cells, tissue remodeling	β-catenin
RASGRP	Calcium and DAG regulated nucleotide exchange factor specific for Ras activation.	Cytoplasm, cytosol, cell membrane, Golgi and peripheral membrane	T and b cell development, homeostasis and differentiation	E2F, FOXO3, PPAR-Y2
HIST1H	Component in chromatin.	Nucleus	Embryonic stem cells	β-catenin
SAMD9L	Urokinase Enhancer factor1. May play a role in tissue injury.	Cytoplasm and nucleus	Proliferation and apoptosis	β-catenin
IFI27	Robust release of cytochrome c from the mitochondria and activation of BAX and caspases 2,3,6,8, and 9	Mitochondrion Membrane protein	Proliferation	STAT1
ANKRD1	Endothelial cell activation. Act as a nuclear transcription factor. Regulates Nanog, Oct4 and POU5F1	Nucleus	Self-renewal and pluripotency in hESCs	TCF regulatable
KCNK15	Putative potassium channel protein	Membrane	Express in HSCs	ND
GADD45	Functions as a regulator of p38 MAPKs by inhibiting p88 phosphorylation and activity.	Nucleus	Apoptosis	Regulates β-catenin transcription
HBEGF	Activator of EGFR family receptor. Neuronal cell survival and proliferation of glial stem cells.	Extracellular space, membrane	Cell proliferation/glial stem cells	Up stream of β-catenin signaling
ABI3BP	ABI family member 3 binding protein. Potentially binds to protein, collagen, glycosaminoglycan and heparin. Interacts with GRB2.	Extra cellular secreted	Lung cancer	TCF dependent signaling
CTGF	IGF-BP8 is a mitogen. Integrin, heparin binding and growth factor activity.	Extracellular space or matrix	Proliferation, differentiation and cell adhesion.	β-catenin

Supplementary Table 2. Identification of β-catenin signaling in EGFR TKI induced drug persistent cells. List of top genes identified as positive regulators and function, physiological role and potentially known transcription factor associated with the target gene expression.
